# Differential expression of PD-L1 between primary and metastatic epithelial ovarian cancer and its clinico-pathological correlation

**DOI:** 10.1038/s41598-021-83276-z

**Published:** 2021-02-12

**Authors:** Sandeep Kumar Parvathareddy, Abdul K. Siraj, Ismail A. Al-Badawi, Asma Tulbah, Fouad Al-Dayel, Khawla S. Al-Kuraya

**Affiliations:** 1 Human Cancer Genomic Research, Research Center, King Faisal Specialist Hospital and Research Center, MBC#98-16, P.O. Box 3354, Riyadh, 11211 Saudi Arabia; 2 Department of Obstetrics-Gynecology, King Faisal Specialist Hospital and Research Center, Riyadh, Saudi Arabia; 3 Department of Pathology, King Faisal Specialist Hospital and Research Centre, P.O. Box 3354, Riyadh, 11211 Saudi Arabia

**Keywords:** Cancer, Biomarkers

## Abstract

Ovarian cancer (OC) is one of the most common gynecologic cancer, which has the worst prognosis and highest mortality rate. The lack of curative treatment and the high relapse rate, especially in advanced OC, continues to present a clinical challenge, highlighting the need for new therapeutic strategies. This study was performed to compare the expression of PD-L1 in primary epithelial ovarian cancer (EOC) and their corresponding peritoneal metastases, as well as to evaluate its correlation with clinico-pathological parameters. In total, 194 treatment naïve paired EOC and peritoneal metastasis were analyzed by immunohistochemistry for PD-L1 expression. Clinico-pathological information was available for all patients. Significant differences in PD-L1 expression were found between primary EOC and peritoneal metastasis (*p* < 0.0001). We found discordant tumor cell PD-L1 expression between primary tumors and corresponding peritoneal metastasis in 34% (66/194) of cases. Furthermore, PD-L1 expression in peritoneal metastasis samples was significantly associated with adverse prognostic factors, such as high proliferative index (Ki67) (*p* = 0.0039) and high histologic grade (*p* = 0.0330). In conclusion, the discordance of PD-L1 expression between primary EOC and corresponding peritoneal metastases suggests that its assessment as a potential biomarker for predicting response to anti-PD-L1 therapy may require analysis of metastatic lesions.

## Introduction

Ovarian cancer (OC) remains the deadliest gynecological malignancy, accounting for ~ 5% of all death from cancer in women^1,2^. Epithelial ovarian cancers (EOC) are the most common histological subtype, comprising > 95% of OCs^3^. Majority of the patients with EOC have advanced stage disease at diagnosis, with metastatic lesions, due to absence of specific clinical symptoms and lack of early screening programs^4–6^. Most currently available treatments are not curative for patients with advanced disease, which could explain the low five-year survival rate of less than 30%^7^. Hence, there is a need for more effective systemic therapies for the management of advanced EOC.

Programmed cell death ligand 1 (PD-L1) has attracted attention as a novel therapeutic target in the context of successful trials in many cancer types^8–10^. PD-1/PD-L1 pathway is considered a critical immune modulatory pathway that inhibits the immune reaction to cancer cells by negatively regulating T-cell functions^11,12^. Blockade of PD-1/PD-L1 signaling pathway using targeted monoclonal antibodies has become a promising therapeutic modality in cancers, with encouraging anti-tumor activity and an increased survival in several cancers^13^. Similarly, it has been shown that PD-L1 inhibitors play an important role in the adjuvant therapy of advanced and treatment-resistant OC^14,15^. Ongoing clinical trials are investigating the efficacy and safety of anti-PD-L1 antibodies in recurrent advanced OC^16,17^.

The immunohistochemical expression of PD-L1 as a prognostic marker and/or predictor of curative effect of anti-PD-L1 therapy has been investigated in various malignancies including OC^18–24^. However, only a few studies have investigated how PD-L1 expression may vary throughout primary tumors or in the primary tumor versus the corresponding metastases^25–28^.

This information can expand the potential predictive value for this biomarker and determine whether the expression of PD-L1 is likely to be more informative in primary tumor tissue or from metastatic site. For this reason, we investigated PD-L1 expression in a series of treatment naïve primary EOC and corresponding peritoneal metastasis. Moreover, we also investigated the correlation between PD-L1 expression status and several important clinico-pathological parameters in EOC from Middle-Eastern ethnicity.

## Results

### Patient characteristics

Median age of the study cohort was 54.5 years (range 19–90 years). High-grade serous carcinoma was the most common histologic subtype, accounting for 64.4% (125/194) of all EOCs. Majority of the patients presented with high FIGO grade (Grade 3–49%; 95/194) and advanced stage (Stage III and IV—91.8%; 178/194) tumors (Table 1).

**Table 1:** Clinico-pathological variables for the patient cohort (n = 194).

	n (%)
**Age**	
Median	54.5
Range	19.0–90.0
**Histopathology**	
High-grade Serous	125 (64.4)
Low-grade Serous	36 (18.6)
Mucinous	13 (6.7)
Endometrioid	14 (7.3)
Clear cell	3 (1.5)
Undifferentiated	3 (1.5)
**FIGO Grade**	
Grade 1	27 (13.9)
Grade 2	66 (34.0)
Grade 3	95 (49.0)
Unknown	6 (3.1)
**pT**	
T1	7 (3.6)
T2	12 (6.2)
T3	175 (90.2)
**pN**	
N0	171 (88.1)
N1	23 (11.9)
**pM**	
M0	153 (78.9)
M1	41 (21.1)
**Stage**	
I	8 (4.1)
II	8 (4.1)
III	137 (70.6)
IV	41 (21.2)
**Residual tumor**	
Present	75 (38.7)
Absent	119 (61.3)

### Distribution of PD-L1 in primary EOC and paired peritoneal metastases

**Figure 1:**
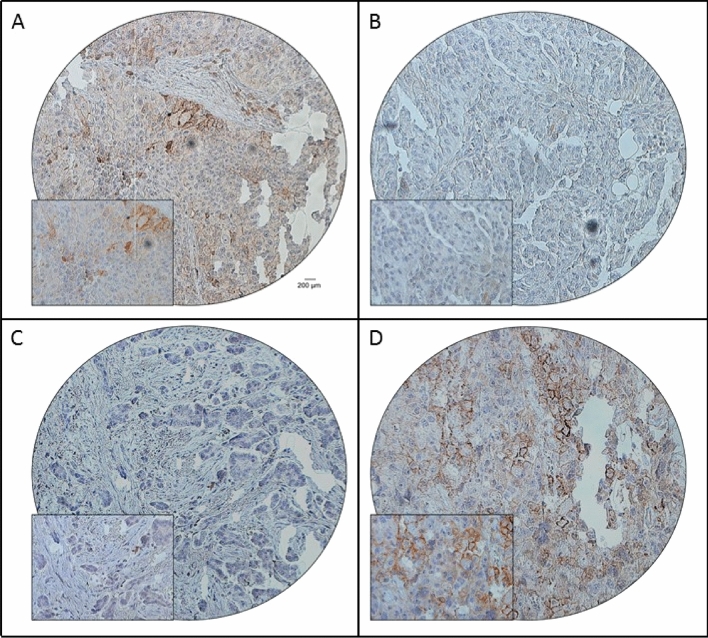
Immunohistochemical analysis of PD-L1 expression in primary EOC and corresponding peritoneal metastasis. EOC array spots showing positive (**A**) and negative (**B**) expression of PD-L1 in primary tumor, with the corresponding peritoneal metastatic tissue showing negative (**C**) and positive (**D**) expression of PD-L1. 20X/0.70 objective on an Olympus BX 51 microscope. (Olympus America Inc, Center Valley, PA, USA) with the inset showing a 40X 0.85 aperture magnified view of the same TMA spot.

PD-L1 expression was analysed in 194 treatment naïve paired primary EOC and peritoneal metastases tissues using tissue microarray (TMA). Positive expression of PD-L1 in primary tumor and matched peritoneal metastases was 32.5% (63/194) and 45.9% (89/194), respectively. Importantly, the difference in expression of PD-L1 between the primary tumor and paired peritoneal metastases was statistically significant (*p* < 0.0001) (Table 2, Fig. 1A–D). Among the 63 cases showing positive PD-L1 expression in primary tumor, 43 also had positive expression of PD-L1 in the paired peritoneal metastasis, whereas 20 cases were negative. Of the 131 cases with negative PD-L1 expression in primary tumor, 85 also had negative expression of PD-L1 in the paired peritoneal metastasis and 46 cases were positive for PD-L1 (Table 2). Thus, the concordance rate of PD-L1 expression was 66.0% (128/194). A discrepancy between the primary tumor and metastatic tissue was noted in 34.0% (66/194) cases.

**Table 2:** Comparison of PD-L1 status between primary EOC and corresponding peritoneal metastases.

Primary tumor	Paired peritoneal metastases			*p* value
	Positive	Negative	Total (%)	
**PD-L1**				
Positive	43	20	63 (32.5)	< 0.0001
Negative	46	85	131 (67.5)	
Total (%)	89 (45.9)	105 (54.1)	194 (100.0)

### Clinico-pathological associations of PD-L1 expression in primary EOC and paired peritoneal metastases

The associations between PD-L1 expression and clinico-pathological parameters was analysed in the primary tumor and their matched peritoneal metastases. In the primary EOCs, positive PD-L1 expression was associated with lymph node metastasis (*p* = 0.0112). PD-L1 expression in metastatic tissues was associated with grade 3 tumors (*p* = 0.0330) and high Ki-67 index (*p* = 0.0039) (Table 3). Interestingly, PD-L1 expression was not associated with mismatch repair deficiency (dMMR) in both primary tumor as well as peritoneal metastases.

**Table 3:** Clinico-pathological associations of PD-L1 protein expression in primary EOC and corresponding peritoneal metastasis.

	Total	Primary EOC			Peritoneal metastasis		
		PD-L1 Positive	PD-L1 Negative	*p* value	PD-L1 Positive	PD-L1 Negative	*p* value
	No. (%)	No. (%)	No. (%)		No. (%)	No. (%)	
No. of patients	194	63 (32.5)	131 (67.5)		89 (45.9)	105 (54.1)	
**Age (Yrs)**							
≤ 50	84 (43.3)	30 (35.7)	54 (64.3)	0.4004	40 (47.6)	44 (52.4)	0.6704
> 50	110 (56.7)	33 (30.0)	77 (70.0)		49 (44.6)	61 (55.4)	
**Histology type**							
High-grade Serous	125 (64.4)	44 (35.2)	81 (64.8)	0.9134	65 (52.0)	60 (48.0)	0.1919
Low-grade Serous	36 (18.6)	9 (25.0)	27 (75.0)		11 (30.6)	25 (69.4)	
Mucinous	13 (6.7)	4 (30.8)	9 (69.2)		4 (30.8)	9 (69.2)	
Endometrioid	14 (7.2)	4 (28.6)	10 (71.4)		6 (42.9)	8 (57.1)	
Clear cell	3 (1.6)	1 (33.3)	2 (66.7)		1 (33.3)	2 (66.7)	
Undifferentiated	3 (1.6)	1 (33.3)	2 (66.7)		2 (66.7)	1 (33.3)	
**FIGO grade**							
Grade 1	27 (14.4)	6 (22.2)	21 (77.8)	0.3354	7 (25.9)	20 (74.1)	0.0330
Grade 2	66 (35.1)	21 (31.8)	45 (68.2)		30 (45.5)	36 (54.5)	
Grade 3	95 (50.5)	35 (36.8)	60 (63.2)		51 (53.7)	44 (46.3)	
**pT**							
T1	7 (3.6)	2 (28.6)	5 (71.4)	0.9731	2 (28.6)	5 (71.4)	0.5963
T2	12 (6.2)	4 (33.3)	8 (66.7)		5 (41.7)	7 (58.3)	
T3	175 (90.2)	57 (32.6)	118 (67.4)		82 (46.9)	93 (53.1)	
**pN**							
pN0	171 (88.1)	50 (29.2)	121 (70.8)	0.0112	75 (43.9)	96 (56.1)	0.1245
pN1	23 (11.9)	13 (56.5)	10 (43.5)		14 (60.9)	9 (39.1)	
**pM**							
pM0	153 (78.9)	50 (32.7)	103 (67.3)	0.9059	72 (47.1)	81 (52.9)	0.5222
pM1	41 (21.1)	13 (31.7)	28 (68.3)		17 (41.5)	24 (58.5)	
**Stage**							
I	8 (4.1)	4 (50.0)	4 (50.0)	0.7473	4 (50.0)	4 (50.0)	0.5408
II	8 (4.1)	3 (37.5)	5 (62.5)		2 (25.0)	6 (75.0)	
III	137 (70.6)	43 (31.4)	94 (68.6)		66 (48.2)	71 (51.8)	
IV	41 (21.2)	13 (31.7)	28 (68.3)		17 (41.5)	24 (58.5)	
**Residual tumor**							
Present	75 (38.7)	29 (38.7)	46 (61.3)	0.1455	35 (46.7)	40 (53.3)	0.8608
Absent	119 (61.3)	34 (28.6)	85 (71.4)		54 (45.4)	65 (54.6)	
**MMR IHC**							
pMMR	185 (98.4)	63 (34.1)	105 (65.9)	0.1158	87 (47.0)	98 (53.0)	0.6332
dMMR	3 (1.6)	0 (0.0)	3 (100.0)		1 (33.3)	2 (66.7)	
**Ki-67 IHC**							
High	111 (59.0)	40 (36.0)	71 (64.0)	0.3769	61 (54.9)	50 (45.1)	0.0039
Low	77 (41.0)	23 (29.9)	54 (70.1)		26 (33.8)	51 (66.2)	
**Progression-free survival**							
Median (months)		14.0	13.0	0.7240	14.0	12.0	0.5245
Range (months)		2.0–87.0	2.0–93.0		3.0–93.0	2.0–92.0	
Median absolute deviation (months)		6.0	7.0		7.0	5.0	

*MMR* mismatch repair, *pMMR* proficient MMR, *dMMR* deficient MMR.

### Prognostic impact of PD-L1 expression in primary EOC and paired peritoneal metastases

**Figure 2:**
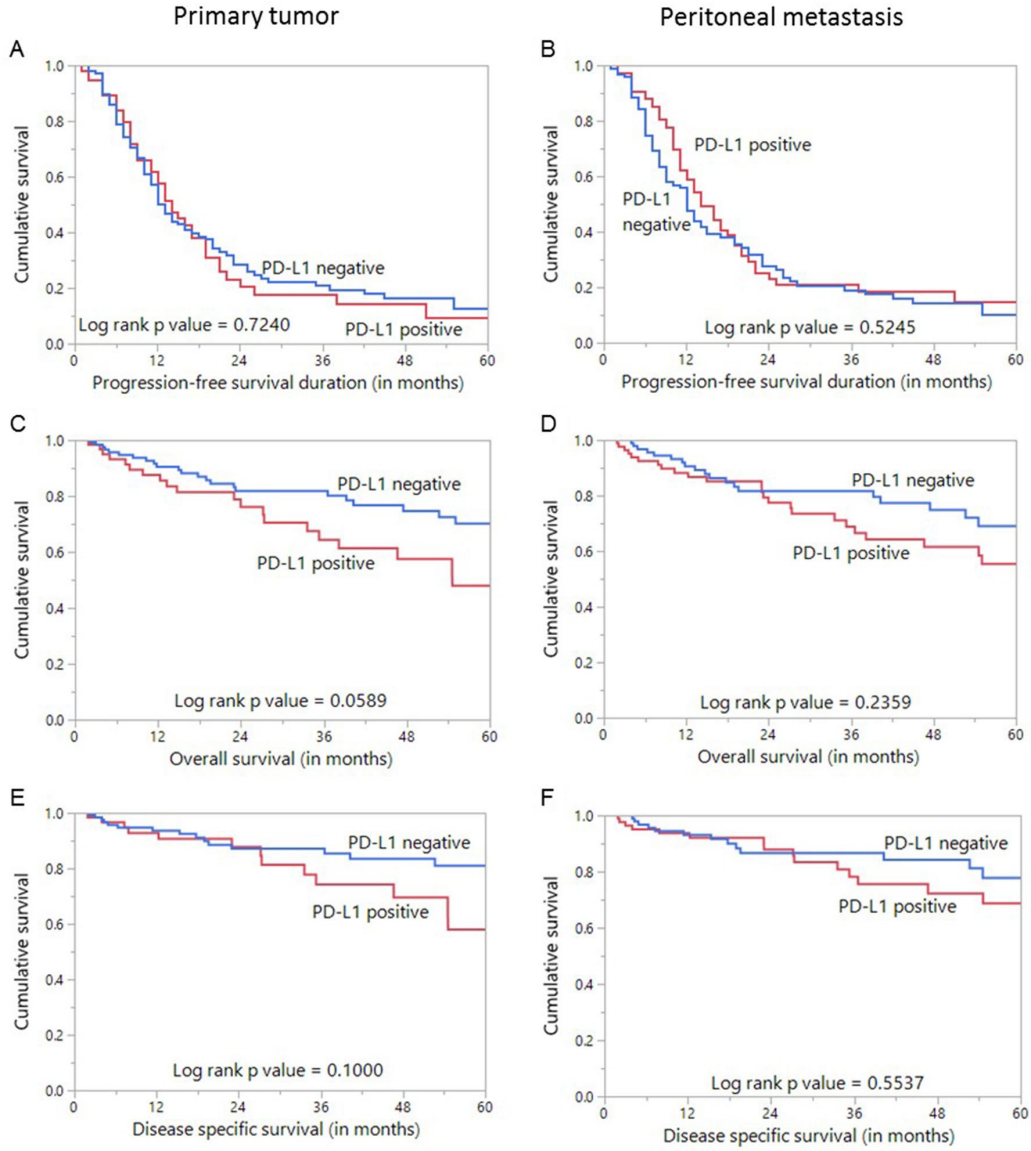
Survival analysis of PD-L1 protein expression in epithelial ovarian cancer. Kaplan Meier survival plot showing no statistically significant difference between PD-L1 positive and negative tumors in both primary and corresponding peritoneal metastases for progression-free survival (**A**,**B**), overall survival (**C**,**D**) as well as disease specific survival (**E**,**F**).

We evaluated the effect of PD-L1 expression on progression-free survival (PFS), overall survival (OS) and disease-specific survival (DSS). PD-L1 expression in both primary tumor and peritoneal metastases was not significantly associated with PFS, OS or DSS (Fig. 2). In the primary EOC tissues, patients with PD-L1 positive tumors (n = 63) had a median follow-up of 22 months (range: 2–153 months) and experienced 43 progression events, whereas patients with PD-L1 negative tumors (n = 131) had a median follow-up of 21 months (range: 2–237 months) and experienced 86 progression events. In the peritoneal metastases tissues, patients with PD-L1 positive tumors (n = 89) had a median follow-up of 22 months (range: 2–237 months) and experienced 54 progression events, whereas patients with PD-L1 negative tumors (n = 105) had a median follow-up of 20 months (range: 2–199 months) and experienced 75 progression events. On multivariate analysis using Cox proportional hazard model for PFS, only stage of tumor was an independent prognostic marker (Table 4).

**Table 4:** Cox regression model analysis for prediction of progression-free survival

Clinico-pathological variables	Progression-free survival			
	Univariate		Multivariate	
	Hazard ratio (95% CI)	*p* value	Hazard ratio (95% CI)	*p* value
Age Above > 50 years (vs ≤ 50 years)	1.13 (0.80–1.61)	0.4795	1.24 (0.85–1.81)	0.2673
Histologic grade High grade (vs. low grade)	1.01 (0.67–1.57)	0.9547	0.83 (0.53–1.33)	0.4304
Lymph node metastasis N1 (vs. N0)	0.95 (0.51–1.63)	0.8651	1.04 (0.52–1.92)	0.8999
Stage IV (vs. I–III)	1.93 (1.25–2.89)	**0.0035**	2.14 (1.34–3.32)	**0.0017**
Residual tumor Present (vs. Absent)	1.25 (0.88–1.77)	0.2141	1.37 (0.94–1.98)	0.0972
PD-L1 (Primary tumor) High (vs. Low)	1.07 (0.73–1.54)	0.7318	1.13 (0.74–1.71)	0.5548
PD-L1 (Peritoneal metastases) High (vs. Low)	0.89 (0.63–1.27)	0.5340	0.93 (0.62–1.38)	0.7180

(Significant *p* values are highlighted in bold)

**Figure 3:**
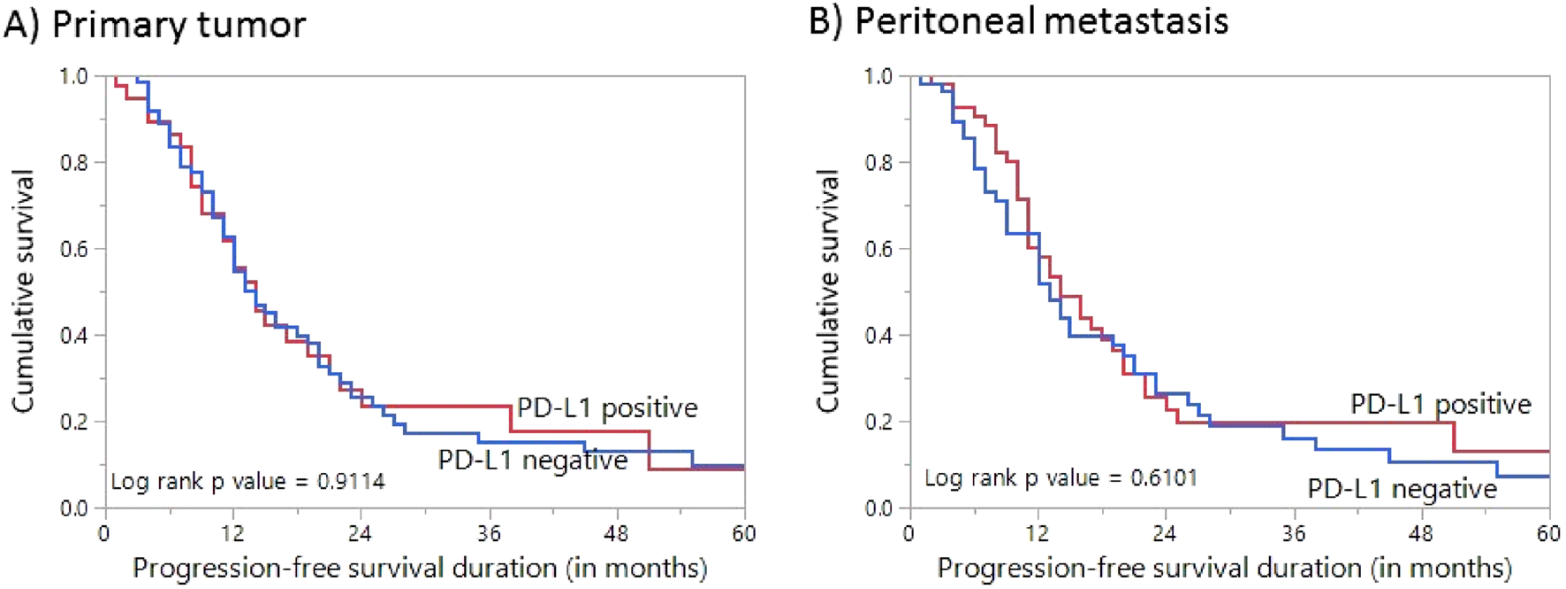
Survival analysis of PD-L1 protein expression in high-grade serous carcinoma. Kaplan Meier survival plot showing no statistically significant difference between PD-L1 positive and negative tumors in both primary (**A**) and corresponding peritoneal metastases (**B**) for progression-free survival.

It is well known that the different histological types of ovarian cancer represent different diseases. Since high-grade serous carcinomas were the predominant histologic subtype in our cohort, we analysed PFS with respect to PD-L1 expression in this subset of patients. Again, PD-L1 expression in both primary tumor and peritoneal metastases was not significantly associated with PFS (Fig. 3).

## Discussion

Very promising results have been obtained with immunotherapeutic agents that target PD-1/PD-L1 pathway^29–32^. PD-L1 is a crucial immune regulatory factor, and as a receptor for PD-1, it plays an important role in the immune escape mechanism of cancer cells^12,33^. It is well known that binding of PD-1 with its ligand, PD-L1 impair T-cell activation and differentiation, and there is evidence that tumor-infiltrating immune cells induce cytokines that upregulate PD-L1 expression^33–35^.

We found PD-L1 positivity in 32.5% (63/194) of primary EOCs. PD-L1 expression was significantly associated with lymph node metastasis. Several previous studies have reported on the clinical associations of PD-L1 expression in ovarian cancer patients, but still have not reached consensus. While some studies found PD-L1 expression in OC to be associated with aggressive clinico-pathological features such as higher tumor stage, grade and poor survival^21,36–38^, others have failed to demonstrate this association^39,40^. Interestingly, a previous meta-analysis has revealed the effect of patients’ ethnicity on prognostic value of PD-L1 expression in OC. Huang et.al. found that PD-L1 expression is a poor prognostic biomarker in Asian population in contrast to the non-Asian patients with OC where PD-L1 is a good prognostic marker^41^. Unfortunately, PD-L1 expression did not affect the progression-free survival in EOC from the Middle-Eastern ethnicity, which could be due to the inherent biases of the study as discussed later. Also, assessment of PD-L1 expression in immune infiltrates, in addition to tumor cells, might provide a clearer picture with regards to prognosis, as shown by previous studies^38,42^. However, use of tissue microarray in our study precluded adequate assessment of PD-L1 expression in immune infiltrates.

Recent studies have highlighted the important role of PD-L1 inhibition in the treatment of OC^43,44^. However, recent evidence has shown that immunohistochemistry (IHC) staining of PD-L1 in OC specimens may not identify all patients who might respond to anti-PD-L1 agents. Indeed, up to 8% of patients with PD-L1 negative tumors were found to have objective response to treatment with anti-PD-L1 agent (Avelumab), whereas many patients who had PD-L1 positive tumors failed to respond^16^. Another Phase I clinical trial in advanced /recurrent ovarian cancer found that only 25% (2 /8) of patients with PD-L1 positive tumors showed response to Atezolizumab^17^. A possible explanation could be the effect of tumor heterogeneity on the predictive value of PD-L1 expression. Given the high tumor heterogeneity in OC, testing for PD-L1 in primary tumors alone may not be an accurate reflection of the biology of metastatic tumors that need to be targeted with immunotherapy. Consistent with this hypothesis, previous reports have found discordance between the primary and metastatic tumors in several cancers such as melanoma, renal cell carcinoma and breast cancer^25–27^.

We compared the PD-L1 expression between primary tumor and peritoneal metastasis to evaluate if intra-patient heterogeneity exists in EOC patients. Peritoneum is usually the initial and most common site of metastasis in OC^45^. The presence of peritoneal metastasis is important for staging, treatment and prognosis of OC patients^46,47^. In our study, we found discordant tumor cell PD-L1 expression between primary tumors and corresponding peritoneal metastasis in a high proportion of cases (34%). Gottlieb and colleagues^28^ also compared the concordance rate of PD-L1 expression in the primary ovarian tumors and their matched metastatic deposits from predominantly treatment naïve high grade serous ovarian carcinoma from 21 patients. In contrast to our study, they found a relatively high concordance of PD-L1 expression (76.2%; 16/21) between the two tissues.

In the present study, we found a higher proportion of peritoneal metastatic tumors showing PD-L1 expression compared to primary EOC (*p* < 0.0001). This suggests the importance of analyzing tissue from metastatic lesions when assessing the predictive value of PD-L1 expression in EOC. Prospective clinical trials might provide further insight and help in selecting patients who could respond to immunotherapy.

Our study also highlights the association between PD-L1 expression and several critical clinico-pathological characteristics in the primary EOC and their matched peritoneal metastasis. We found PD-L1 expression to be associated with aggressive markers such as lymph node metastasis in primary EOC and high Ki-67 index and high grade tumors in metastatic tissues. Although no significant correlation was observed between PD-L1 expression and clinical outcome, the significant association with aggressive clinico-pathological parameters might indirectly suggest a similar association. Furthermore, although studies have previously shown an association between PD-L1 expression and microsatellite instability status^48,49^, we did not find a similar association in our cohort. This could be partly explained by the very low incidence of dMMR in our cohort (1.6%; 3/194). Also previous studies have shown dMMR to be more common in clear cell OC and associated with PD-L1 in this subset of OC^48,50^, whereas our cohort had only three cases of clear cell carcinoma. This may have contributed to the lack of association between PD-L1 and dMMR in our study.

While this study provides important information with potential impact in clinical practice for EOC from Middle Eastern ethnicity, it has a few limitations. Firstly, patients were enrolled over a long period of time (28 years), during which the surgical and therapeutic approach may have changed, leading to treatment bias. Secondly, preserved antigenicity and better fixation of peritoneal implants could be a technical confounder leading to higher expression of PD-L1 in peritoneal metastases. However, this is more pronounced in biopsy samples, whereas all our peritoneal metastasis samples were surgically resected specimens, which mitigates this confounding effect to an extent. Thirdly, PD-L1 expression should have ideally been assessed both on tumor cells and immune infiltrates. However, our focus was on the differential expression of PD-L1 between primary tumor and corresponding peritoneal metastases. Hence, PD-L1 expression in immune infiltrates was not assessed.

In conclusion, the discordance in PD-L1 expression between the primary EOC and the matched peritoneal metastasis observed in our study suggests that testing for PD-L1 expression in both metastatic tumors and primary EOC could increase the predictive role of PD-L1 for responders to immunotherapy in these patients.

## Methods

### Sample selection

One-hundred and ninety-four EOC patients diagnosed between 1989 to 2017 at King Faisal Specialist Hospital and Research Center (Riyadh, Saudi Arabia) with available primary and peritoneal metastases archival tissue samples were included in the study. All the patients were treatment-naïve. Primary tumor samples and the corresponding peritoneal metastases were collected at the same time for all the cases. Clinico-pathological data were collected from case records, the details of which are summarized in Table 1. Progression-free survival was computed from date of surgery for patients who underwent primary cytoreduction to date of disease progression or death from any cause. The median follow-up time was 21 months (range, 2–237 months). Tumors were classified according to WHO Classification of female genital tumors (2020). International Federation of Gynecology and Obstetrics (FIGO) system was used for staging and grading of tumors.

### Ethics declarations

Institutional Review Board of King Faisal Specialist Hospital and Research Centre provided ethical approval for the current study. Research Advisory Council (RAC) granted waiver of informed consent for use of retrospective patient case data under project RAC# 2190 015. All the methods were carried out in accordance with relevant guidelines and regulations.

### Tissue microarray (TMA) construction and immunohistochemistry (IHC)

Tissue microarray (TMA) format was utilized for immunohistochemical analysis of the EOC samples. TMA was constructed as previously described^51^. Briefly, modified semiautomatic robotic precision instrument (Beecher Instruments, Woodland, WI) was used to punch tissue cylinders with a diameter of 0.6 mm from representative tumor area of the donor tissue block and brought into the recipient paraffin block. Two 0.6-mm cores of EOC were arrayed from each case.

Tissue microarray slides were processed and stained manually as described previously^52^. Primary antibody against PD-L1 (E1LN3, 1:100 dilution, pH 9.0, Cell Signaling Technology, Danvers, MA) was used. A membranous and/or cytoplasmic staining was observed. Only the membrane staining was considered for scoring. PD-L1 was scored as described previously^39^. Briefly, the proportion of positively stained cells was calculated as a percentage for each core and the scores were averaged across two tissue cores from the same tumor to yield a single percent staining score representing each cancer patient. For the purpose of statistical analysis, the scores were dichotomised. Cases showing expression level of ≥ 5% were classified as positive and those with less than 5% as negative.

Mismatch repair (MMR) protein as well as Ki-67 staining and evaluation was done as described previously^53,54^. MMR protein expression was evaluated using MSH2, MSH6, MLH1 and PMS2 proteins. Tumor was classified as deficient MMR (dMMR) if any of the four proteins showed loss of staining in cancer with concurrent positive staining in the nuclei of normal epithelial cells. Otherwise, they were classified as proficient MMR (pMMR). For Ki-67, nuclear staining was considered as positive. The cutoff for high Ki-67 was taken as more than 30% of tumor nuclei staining in the total tumor area.

IHC scoring was done by two pathologists, blinded to the clinico-pathological characteristics. Discordant scores were reviewed together to achieve agreement.

### Statistical analysis

The associations between clinico-pathological variables and protein expression was performed using contingency table analysis and Chi square tests. Mantel-Cox log-rank test was used to evaluated progression-free survival. Two-sided tests were used for statistical analyses with a limit of significance defined as *p* value < 0.05. Data analyses was performed using the JMP11.0 (SAS Institute, Inc., Cary, NC) software package.
